# Direct anodic hydrochloric acid and cathodic caustic production during water electrolysis

**DOI:** 10.1038/srep20494

**Published:** 2016-02-05

**Authors:** Hui-Wen Lin, Rocío Cejudo-Marín, Adriaan W. Jeremiasse, Korneel Rabaey, Zhiguo Yuan, Ilje Pikaar

**Affiliations:** 1 The University of Queensland, Advanced Water Management Centre (AWMC), QLD 4072, Australia; 2 Photovoltaics and Optoelectronic Devices Group, Physics Department, Universitat Jaume I, 12071 Castelló, Spain; 3 MAGNETO special anodes B.V., Calandstraat 109, 3125 BA Schiedam, The Netherlands; 4 Laboratory of Microbial Ecology and Technology (LabMET), Ghent University, Coupure Links 653, 9000 Ghent, Belgium; 5 The University of Queensland, The School of Civil Engineering, QLD 4072, Australia

## Abstract

Hydrochloric acid (HCl) and caustic (NaOH) are among the most widely used chemicals by the water industry. Direct anodic electrochemical HCl production by water electrolysis has not been successful as current commercially available electrodes are prone to chlorine formation. This study presents an innovative technology simultaneously generating HCl and NaOH from NaCl using a Mn_0.84_Mo_0.16_O_2.23_ oxygen evolution electrode during water electrolysis. The results showed that protons could be anodically generated at a high Coulombic efficiency (i.e. ≥ 95%) with chlorine formation accounting for 3 ~ 5% of the charge supplied. HCl was anodically produced at moderate strengths at a CE of 65 ± 4% together with a CE of 89 ± 1% for cathodic caustic production. The reduction in CE for HCl generation was caused by proton cross-over from the anode to the middle compartment. Overall, this study showed the potential of simultaneous HCl and NaOH generation from NaCl and represents a major step forward for the water industry towards on-site production of HCl and NaOH. In this study, artificial brine was used as a source of sodium and chloride ions. In theory, artificial brine could be replaced by saline waste streams such as Reverse Osmosis Concentrate (ROC), turning ROC into a valuable resource.

Hydrochloric acid (HCl) and caustic soda (NaOH) are both widely used chemicals for water and wastewater treatment^1^,^2^. Caustic is mainly produced in the chlor-alkali process by the electrolysis of sodium chloride (NaCl) with concomitant chlorine production^2^,^3^. Although HCl cannot be directly synthesized in this process, it can be formed by burning chlorine and hydrogen gas produced in the cathode^3^. However, the transport, storage and handling of concentrated HCl and NaOH come with serious occupational health and safety (OH&S) concerns for the water industry. As in most cases both compounds are used at relatively low concentrations by the water industry, there is a general interest in on-site generation of moderate strength HCl and NaOH solutions to avoid the aforementioned issues. On-site generation would also avoid the concentration step and thus reduce the overall energy consumption.

Protons (H^+^) and hydroxide ions (OH^−^) could be produced by electrolysis of water using a two-chambered electrochemical cell with anode being fed with NaCl containing water and cathode fed with clean water. However, the currently commercially available anode materials such as mixed metal oxide (MMO) coated titanium and boron doped diamond (BDD) are prone to chlorine formation even at low chloride concentrations^4^,^5^,^6^. Consequently, these materials do not allow for the direct production of HCl from NaCl solutions.

To avoid chlorine formation, a five-compartment electrochemical system (i.e. bipolar membrane electrodialysis) was proposed for simultaneous acid and caustic generation from reverse osmosis concentrates^7^. While the feasibility of simultaneous production of acid and caustic was demonstrated, the practical and economic feasibility is expected to be limited due to complex reactor configuration and large energy requirements of the system caused by the use of multiple membranes.

Previous studies showed that coating of titanium electrodes with manganese–molybdenum oxides instead of Ir MMO remarkably decreased the electrocatalytic activity towards formation of hypochlorite^8^,^9^,^10^,^11^. Whereas these studies aimed to generate hydrogen from seawater under either mild alkaline or acidic conditions using undivided electrochemical cells, the results suggest that this material could potentially prevent chlorine formation during the production of hydrochloric acid at moderate strengths. Indeed, it has been hypothesized that MnO_2_ based coatings can act as a diffusion barrier to chloride ions. This enables the formation of a high degree of concentration polarization, thus increasing the concentration overpotential for the chlorine evolution reaction. Consequently, oxygen evolution from water oxidation is favoured^12^. In this work, we therefore hypothesize that without the occurrence of anodic chlorine formation, it would be feasible to use the Mn_x_Mo_y_O_z_ anode to simultaneously produce HCl and NaOH without the necessity for two additional bipolar membranes and deionized water as media in the above-mentioned electrochemical system^7^. Hence, our proposed system can operate at a much lower ohmic resistance and thus consumes less power.

Here, we aim to evaluate the feasibility of using Mn_x_Mo_y_O_z_ anodes for simultaneous HCl and NaOH production using a three-compartment electrochemical cell. In this configuration, the anode and middle compartments are separated by an anion exchange membrane (AEM) and the cathode and middle compartment are separated by a cation exchange membrane (CEM) in which a concentrated NaCl solution is recirculated over the middle compartment. In this way, HCl and NaOH can be produced simultaneously in the anode and cathode compartment, respectively. In theory, not only artificial brine but also saline (waste) streams (e.g. reverse osmosis concentrates) could be used and could thus potentially revolutionize the remediation of saline waste streams.

## Results and Discussion

### Electrode performance

The first set of experiment showed that the average CE for HCl generation was 65.0 ± 3.5% at a final HCl strength of 0.81 ± 0.04 wt%, while the average CE for caustic generation was 88.6 ± 0.5% at a final caustic strength of 1.16 ± 0.01 wt%. The final pH levels in the anode and cathode compartments were 0.95 ± 0.04 and 13.63 ± 0.03, respectively. IC analysis of the chloride concentrations in the anode also confirmed the HCl production (0.78 ± 0.05 wt%).

The reduction in CE for HCl production was due to proton cross-over through the AEM to the middle compartment and the reduction in CE for NaOH production was caused by hydroxide back-diffusion through the CEM to the middle compartment, and possibly also proton cross-over through the CEM to the cathode compartment. The protons in the middle compartment neutralise hydroxide ions migrated through the CEM and may also migrate through the CEM to the cathode compartment. The final pH of the middle compartment decreased over time to 2.0 ± 0.0, indicating proton cross-over from anode was more pronounced than hydroxide back-diffusion from cathode. The net increase of proton concentration in the middle compartment (pH at 2.0) accounted for 10.8 ± 0.1% of the total charge applied. The CE loss for hydroxide production was estimated at 11.4 ± 0.5%. Hence, the total CE loss for HCl production is estimated to be 22.2 ± 0.5% of the total charge applied. Based on ion exchange capacity of the AEM (total ion capacity: 1.3 meq/g; weight of dry membrane contacted with solution: 1.44 g) and CEM (total ion capacity: 1.6 meq/g; weight of dry membrane contacted with solution: 1.64 g) used in the experiments, the estimated CE loss for H^+^ was calculated at 2.1% and 2.9% for the AEM and CEM, respectively. Importantly, the observed chlorine formation only accounted for 5.3 ± 2.0% of the charge supplied. Considering the above-mentioned factors, the final electron balance for anodic reactions equalled 97.6 ± 2.0%. In addition, the anode potential and cell voltage were 1.56 ± 0.02 V versus NHE, and 5.4 ± 0.0 V, respectively, clearly showing the reproducibility of the experiments. Considering the standard potential E^0^ for oxygen evolution of 1.17 V versus NHE (at a pH of 1), the overpotential for oxygen evolution can be estimated at 0.39 V. This value is similar to overpotentials found for other known catalytic coatings for oxygen evolution, as discussed in detail in Frydendal, *et al.*^13^. The reduction in cell voltage could be further achieved by using a better reactor configuration or membranes having a smaller area resistance.

In the second set of experiments (n = 3), a bicarbonate buffer solution was used as the anolyte instead of 0.1 wt% HCl solution to confirm the affinity towards oxygen evolution rather than chlorine formation. The observed CE for free available chlorine (i.e. the sum of chlorine, hypochlorous acid and hypochlorite ion) generation was as low as 2.9 ± 0.6% at pH ~ 7.5, indicating the high affinity for oxygen evolution with CE for water oxidation above 97%. This high affinity towards oxygen evolution is in agreement with our chlorine evolution test (see Supplementary Information). In addition, the average CE for NaOH generation was 86.2 ± 4.3%. The pH of the middle compartment increased over time to 11.7 ± 0.6, which accounted for 9.3 ± 7.4% of the total charge applied. The results confirm that the CE loss for NaOH generation was mainly due to hydroxide back-diffusion from the cathode to the middle compartment.

### Implications for practice

Here, we showed the feasibility of simultaneous production of HCl and NaOH through the use of Mn_0.84_Mo_0.16_O_2.23_ coated titanium electrodes. This was achieved with a three-compartment electrochemical cell with artificial brine as the solution in the middle compartment. The method presented here marks a major step forward towards on-site production of HCl and NaOH. The use of such a technology would eliminate the OH&S concerns associated with the transport, storage and handling of concentrated HCl and NaOH. In this study, we used NaCl solution as a source of sodium and chloride ions. In practical applications, NaCl solution could be replaced by saline waste streams such as Reverse Osmosis Concentrate (ROC) or seawater, which potentially can revolutionize the treatment of saline waste streams like ROC by turning ROC into a valuable resource instead of a waste stream.

In this proof-of-concept study, HCl and NaOH were produced at strengths of 0.81 ± 0.04 wt% and 1.16 ± 0.01 wt%, respectively. For a practical situation, a further increase in solution strengths (e.g. 3 ~ 5 wt%) are recommended in order to minimize the required storage volume.

The stability of the coating under the conditions applied should be tested during long-term experiments and accelerated life time tests, whereas the oxygen efficiency of the coating may be further improved through addition of other metals in the coating (e.g. Tungsten), and improving the anodic application process by repeated anodic deposition^14^. Furthermore, other factors that potentially hinder the industrial implementation such as the degradation of the electrode by the oxide growth on the substrate, should also be investigated^10^. Indeed, future research is warranted to investigate the effect of the supporting electrolyte on chloride oxidation on Mn_x_Mo_y_O_z_ coatings in detail.

In addition, the AEM used in this study was prone to significant proton cross-over, thus the process efficiency could be further enhanced by using membranes being less prone to proton cross-over, such as membranes used as acid blocker^15^. Due to the potential impact of chlorine on anion exchange membranes, chlorine resistant membrane or porous plate separators could be suggested^16^,^17^.

## Methods

### Electrochemical cell and operation

**Figure 1:**
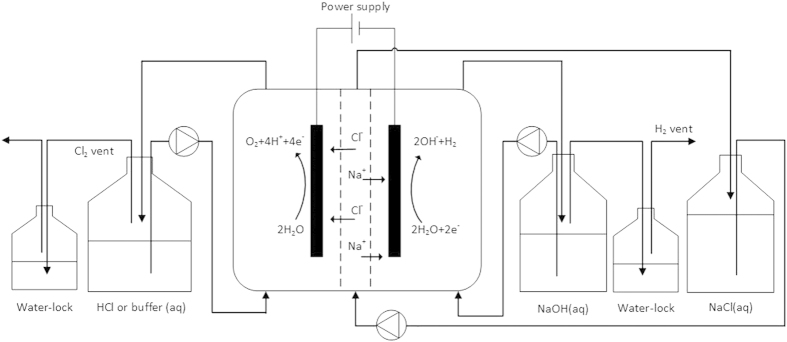
Schematic overview of the experimental setup.

The methods used for the electrode preparation and characterization are described in detail in Supplementary Information^9^,^18^,^19^. Figure 1 presents a schematic overview of the experimental setup. The three-compartment electrochemical cell consisted of three Perspex frames with internal dimensions of 20 × 5 × 1 cm creating volumes of 100 mL for each compartment. An AEM (Ultrex AMI-7001, Membranes International Inc., USA) separated the anode from the middle compartment while the cathode and middle compartments were separated by a CEM (Ultrex CMI-7000, Membranes International Inc., USA). The produced mesh shaped Mn_0.84_Mo_0.16_O_2.23_ coated titanium electrode and a stainless steel mesh (6 mm mesh size, 0.8 mm wire) with a 24 cm^2^ projected surface were used as the anode and cathode material, respectively. All solutions (i.e. anode, cathode and middle solutions) were recirculated at a flow rate of 4 L/h using a peristaltic pump. An Ag/AgCl reference electrode (assumed at + 0.197 V versus NHE) was used in the anode compartment. Experiments were galvanostatically controlled at a current density of 250 A/m^2^ using a Wenking potentiostat/galvanostat (KP07, Bank Elektronik, Germany). Electrode potentials were recorded every 2 minutes using a data acquisition unit (Agilent Technologies, USA). Water-locks were used in the anode and cathode compartments to prevent oxygen (anode) and hydrogen gas (cathode) build-up. A 200 mL caustic solution (2 wt%) was used as the anode water-lock to trap any chlorine gas formed.

### Procedures of experimental runs

Preliminary results showed that the prepared Mn_0.84_Mo_0.16_O_2.23_ coated titanium electrode had a much lower affinity towards chlorine evolution than commercially available Ta/IrO_x_ coated titanium electrodes (see Supplementary information). Subsequently, two sets of 4-hour experimental runs were conducted. The first set of experiments (n = 3) was conducted to determine the efficiency in terms of HCl and NaOH production. A 300 mL HCl solution (1 g/L) was used as the anolyte and a 300 mL NaOH (1 g/L) was used as the catholyte to provide sufficient initial conductivity. A 1L NaCl solution (35 g/L) was used in the middle compartment. At the end of each experiment, liquid samples from the anode and cathode were taken for measurements of HCl and NaOH, respectively. The second set of experimental runs (n = 3) was performed to confirm that chlorine formation was negligible. A 1 L solution of 40 g/L NaHCO_3_ and 1 g/L NaCl was used as the anolyte and a 500 mL NaOH solution (1 g/L) was used as catholyte. A 1L NaCl solution (35 g/L) was used in the middle compartment. The NaHCO_3_ solution was used to maintain its anodic pH level > 7.5, thus any formed molecular chlorine would remain in the solution as hypochlorous acid and hypochlorite ion rather than chlorine gas. As such, the CE for chlorine formation can be determined accurately. Liquid samples from the cathode were taken for measurements of NaOH production after 4-hour operation. At the end of each experiment, liquid samples from the anode were taken for measurement of the chloride and chlorine concentrations and the final pH values of all compartments were also measured.

### Chemical analyses

The HCl and NaOH concentrations were determined by titration using a 1 M NaOH or a 1 M HCl solution, respectively. pH values were measured using a hand-held pH meter (Eutech Instruments, Australia). Total and free chlorine were measured by DPD free chlorine test kits (Hach Lange, Germany). These test kits are based on the DPD – photometric method, which is in line with standard methods (USEPA Standard method 4500-CI G). Chloride concentration was measured using Ion Chromatography equipped with a Dionex 2010i system.

## Additional Information

**How to cite this article**: Lin, H.-W. *et al.* Direct anodic hydrochloric acid and cathodic caustic production during water electrolysis. *Sci. Rep.* **6**, 20494; doi: 10.1038/srep20494 (2016).

## Supplementary Material

Supplementary Information
