# Supplementary material for: Direct anodic hydrochloric acid and cathodic caustic production during water electrolysis
Source: Sci Rep. 2016 Feb 5;6:20494. doi: 10.1038/srep20494 (PMC4742805; doi:10.1038/srep20494)
Supplement: Supplementary Information [file srep20494-s1.pdf]

Supplementary Information

Direct anodic hydrochloric acid and cathodic caustic production during water electrolysis

Authors: Hui-Wen Lin, Rocío Cejudo-Marín, Adriaan W. Jeremiasse, Korneel Rabaey, Zhiguo Yuan and Ilje Pikaar*

**Correspondence should be addressed to:* Ilje Pikaar, *The School of Civil Engineering, The University of Queensland, St. Lucia, QLD 4072, Australia*

Phone: +61 7 3345 1389; E-mail: i.pikaar@uq.edu.au

Electrode preparation and characterization

Flattened titanium (Ti) mesh was used as electrode substrate. The Ti substrate was coated with an Ir MMO undercoating (MAGNETO CS13-0522) using the paint-thermal decomposition method[18]. The $Mn_xMo_yO_z$ coating was then applied on the Ir MMO undercoating using the anodic deposition method[9]. First, the Ir MMO coated Ti was activated for 10 minutes at an anodic current density of 1000 A/m^2 in 1M H_2SO_4 solution. Next, $Mn_xMo_yO_z$ was anodically deposited for 60 min at 600 A/m^2 from a deposition bath (pH 0, 90°C) which contained 0.2 M $MnSO_4$ and 0.003 M Na_2MoO_4. Electrode characterization (n=2) was done using $Mn_xMo_yO_z$ coated Ti plates prepared following the same procedures as the titanium mesh and addressed from a structural and crystallographic point of view. Structural characterization was performed using a field emission scanning electron microscope (JEM-3100F, JEOL, USA), and the crystallographic structure of the deposits was examined by X-ray diffraction using Cu Kα radiation (D5000 diffractometer, Siemens).

SI Fig. 1 shows the structural characterization of the anodically deposited $Mn_xMo_yO_z$ coatings. SI Fig. 1(a) reveals the discontinuous nature of coatings, showing a typical mud-crack structure[19]. SI Fig. 1(b) shows the cross-sections of coatings, indicating good similarity in coating thickness (around 5-7 µm). The EDS compositional analyses revealed that a good compositional homogeneity of the coatings was achieved with a coating composition of $Mn_{0.84}Mo_{0.16}O_{2.23}$ (SI Fig. 2).

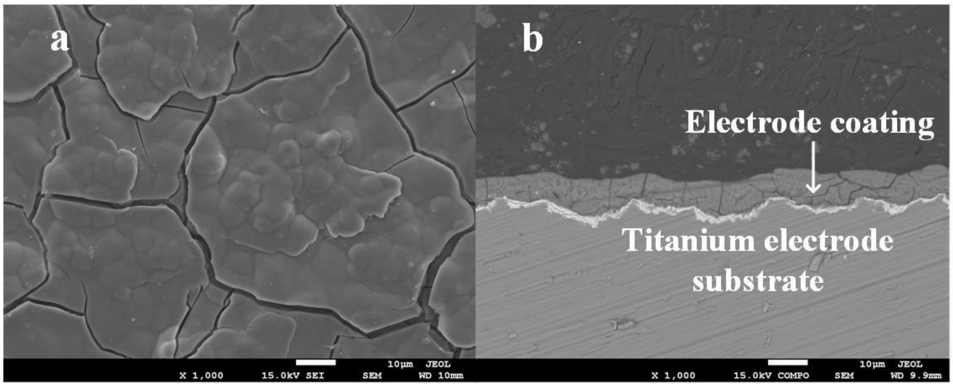

SI Figure 1. Typical example of (a) Top view and (b) Cross-section view of the $Mn_{0.84}Mo_{0.16}O_{2.23}$ coating.

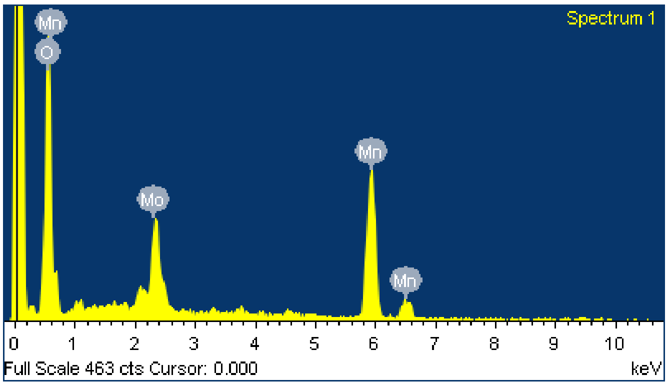

SI Figure 2. Representative spectrum of $Mn_xMo_yO_z$ coated titanium plate electrodes. Electrode samples were analysed by energy-dispersive X-ray spectroscopy. Result clearly **indicates the presence of Mn and Mo in the film. Besides the diffraction peaks for the substrate (Titanium), no other relevant peak was identified, indicating the amorphous nature of the coatings.**

Chlorine evolution test

$Mn_{0.84}Mo_{0.16}O_{2.23}$ and Ta/IrO_x electrodes were used as anodes while uncoated titanium was used as the cathode material. Each experiment was carried out for 20 minutes in duplicate at a 500 A/m^2 fixed current density in an undivided cell using a 25g/L NaCl solution (0.8 L) as the electrolyte at 20 ºC. Mixing (100 rpm) was provided to maintain well-mixed condition. The chlorine/ hypochlorite concentration was determined at the end of each experiment.

Experiments showed that the prepared $Mn_{0.84}Mo_{0.16}O_{2.23}$ electrode had a much lower CE for chlorine evolution than Ta/IrO_x coated titanium electrodes (i.e. 3.8±1.8% versus 86.8±1.3%) and could thus potentially be used for efficient anodic HCl and cathodic NaOH generation.
